# The Smart Life Stay (SLS) program: effects of a lifestyle intervention program in combination with health tourism and health guidance for type 2 diabetes

**DOI:** 10.1038/s41387-020-00136-x

**Published:** 2020-08-29

**Authors:** Madoka Matsushita, Akiko Muramoto, Eri Nomura, Yukari Eguchi, Ayako Kato, Yoshiko Sano, Mai Kabayama, Masashi Arakawa, Yuko Oguma, Daisuke Yabe, Masaaki Matsunaga, Hiroshi Yatsuya, Hiroshi Arima, Kazuyo Tsushita

**Affiliations:** 1 Comprehensive Health Science Center, Aichi Health Promotion Public Interest Foundation, 1-1, Gengoyama, Morioka, Higashiura-cho, Chita-gun, Aichi-ken 470-2101 Japan; 2 Kanagawa University of Human Services, Faculty of Health & Social Services, School of Nutrition & Dietetics, 1-10-1 Heisei-cho, Yokosuka-shi, Kanagawa-ken, 238-8522 Japan; 3 Division of Health Sciences, Osaka University Graduate School of Medicine, 1-7 Yamadaoka, Suita-shi, Osaka-fu 565-0871 Japan; 4 Health Tourism Research Fields, Graduate School of Tourism Sciences University of the Ryukusu, 1 Senbaru, Nishihara-cho, Nakagami-gun, Okinawa-ken, 903-0129 Japan; 5 Sports Medicine Research Center & Graduate School of Health Management, Keio University, 4-1-1 Hiyoshi, Kohoku-ku, Yokohama, Kanagawa-ken 223-8521 Japan; 6 Department of Diabetes and Endocrinology, Gifu University Graduate School of Medicine, 1-1 Yanagido, Gifu-shi, Gifu-ken 501-1194 Japan; 7 Department of Public Health, Fujita Health University School of Medicine, 1-98 Dengakugakubo, Kutsukake-cho, Toyoake-shi, Aichi-ken 470-1192 Japan; 8 Department of Endocrinology and Diabetes, Nagoya University Graduate School of Medicine, 65 Tsurumai-cho, Shouwa-ku, Ngoya-shi, Aichi-ken 466-8550 Japan

**Keywords:** Preventive medicine, Clinical trials

## Abstract

**Background:**

The aim of this study was to determine the effectiveness of the Smart Life Stay (SLS) program, which is an experience-oriented stayover program, in combination with health tourism and mandatory health guidance on glucose metabolism after 2 years.

**Methods:**

The participants of the SLS program (*n* = 792) were recruited from a database of 23 medical insurers. They underwent a mandatory health examination termed Specific Health Checkups in 2014. The participants were included if they had diabetes or were at a high risk of diabetes and if they satisfied the following inclusion criteria: (1) body mass index (BMI; kg/m^2^) > 25, or (2) waist circumference (WC; cm) > 85 for men and > 90 for women, or (3) hemoglobin A1c (HbA1c; %) > 5.6, or (4) fasting plasma glucose (FPG; mg/dl) > 100. Individuals who corresponded to one or more items were included as study participants. The control subjects (*n* = 3645) were nonparticipants of the program who were selected from the database and met the inclusion criteria. The lifestyle changes and changes in mean BMI, WC, FPG, and HbA1c in both groups from baseline to 2-year follow-up were compared by inverse probability weighting of a propensity score.

**Results:**

The percentage of people who exercised regularly increased significantly in the SLS group compared with the control group. In the SLS group, BW, BMI, and WC significantly decreased by 1.75 kg, 0.60 kg/m^2^, and 1.45 cm, respectively, whereas in the control group, WC, FPG, and HbA1c increased significantly by 0.38 cm, 3.37 mg/dl, and 0.12%, respectively. The comparison between groups revealed that the BW, BMI, WC, FPG, and HbA1c improved significantly in the SLS group.

**Conclusions:**

The SLS program is suggested to help improve glucose metabolism. This program could be a feasible option as a lifestyle intervention program for diabetes.

## Introduction

The number of adults suffering from diabetes was estimated to be 463 million in 2019; by 2045, this number will increase to 700 million^1^. Due to medical costs and loss of work, diabetes and its complications result in huge economic losses, not only for patients but also for national economies^2^. Numerous studies have reported that lifestyle interventions, such as a combination of diet and physical activities, are effective for the prevention of diabetes and its complications^3–5^. Therefore, the establishment of more feasible diabetes management strategies is an urgent global health issue.

In Japan, since 2008, the Japanese government has required public medical insurers to conduct medical checkups and provide guidance referred to as Specific Health Checkups (SHC) and the Specific Health Guidance (SHG)^6^ for insured patients aged 40–74 years. This is one of the government’s strategies to prevent diabetes. Under this system, the medical insurers maintain a nationwide unified database of medical checkups of all insured persons. In addition, based on the results of the medical checkups, individuals with metabolic syndromes receive guidance on weight reduction. Numerous studies have already shown that this guidance is effective for weight reduction and glycemic control^7–11^ and it is also cost-effective^12^. However, the SHG was found to be insufficient^6^ for some subjects. In addition, it included patients with obesity as targeted subjects but excluded nonobese patients with diabetes and preliminary diabetes. Because nonobese patients are more common in Asian populations^13^, more effective strategies for those with diabetes and those at high risk of diabetes with or without obesity need to be established.

The health tourism industry has been gaining a growing global interest^14^. It provides medical treatment, relaxation, balanced diet, exercise, and cosmetic services, resulting in its growing popularity worldwide. The health tourism industry, which uses rich local and national resources, such as hot springs and natural beauty, has begun to benefit local economies; however, only a few programs have examined the effects of the health tourism on improving health.

Therefore, a new lifestyle intervention program that combines health guidance and health tourism was developed. The Smart Life Stay (SLS) program is an experience-oriented health tourism program for patients with diabetes or those at a high risk of diabetes. In this program, the patients are treated like customers who want to take part in a holiday and health improvement program.

The aim of the study was to report the 2-year follow-up results of the program on participants’ glucose metabolism and other metabolic parameters.

## Materials and methods

### Study design and subjects

The study adhered to the statement of the Transparent Reporting of Evaluations with Nonrandomized Designs (TREND)^15^. Propensity score analysis was employed to examine the effectiveness of the SLS program. The SLS program participants were recruited (the SLS group: *n* = 792) from a database of 23 medical insurers in 2014. They underwent SHC, such as those from certain communities and workplaces.

The participants were included if they had diabetes or were at a high risk of diabetes^16^ and if they satisfied the following criteria: (1) body mass index (BMI; kg/m^2^) > 25, or (2) waist circumference (WC; cm) > 85 for men and > 90 for women, or (3) hemoglobin A1c (HbA1c; %) > 5.6, or (4) fasting plasma glucose (FPG; mg/dl) > 100. Individuals who corresponded to one or more items were included as study participants. The exclusion criteria included a history of myocardial infarction or cerebral infarction, systolic blood pressure (SBP) > 160 mmHg or diastolic blood pressure (DBP) > 100 mmHg, diabetes with poor glycemic control^17^ or critical diabetic complications, current treatment for depression and schizophrenia, history of anaphylaxis caused by food allergies^18^, alcoholism^19^, and dementia^20^. The control subjects (*n* = 3645) were nonparticipants of the program who were selected from the same database using the same inclusion criteria. The study protocol was conducted by the Comprehensive Health Science Center Aichi Health Promotion Public Interest Foundation and has been approved by the Ethics Review Committee of the center. Other study protocols were approved by the Ethics Review Committee of the Japan Association for Diabetes Education and Care. The organizations explained the study to the subjects and obtained their written consent to participate.

### Intervention

**Fig. 1:**
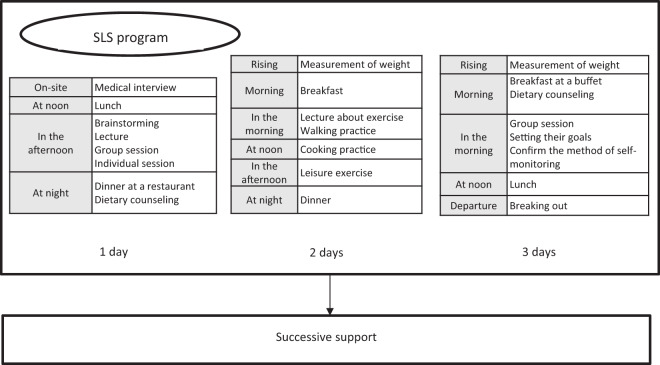
An example of the SLS program. SLS Smart Life Stay program group.

The core program of the intervention was based on the SHG in Japan^21^. The participants were briefed about their medical condition and lifestyle through the results of their medical checkups. In addition, they were instructed to set their lifestyle improvement goals at the initial counseling. The intervention was adapted to the individual participant’s medical background and lifestyle. Health-care professionals, such as physicians, public nurses, fitness trainers, and registered dietitians, performed the intervention between April 2015 and March 2016 at resort hotels, community centers, or spas that were affiliated with the recruitment medical insurers. These professionals received training on the intervention methods before the program initiation. The components of the intervention were (1) a stayover program using the local tourism industry, (2) an experience-oriented diet and exercise program, and (3) a program that includes group sessions (Fig. 1). The program duration and group size differed between each of the 23 recruitment medical insurers and were based on the capacity of their facilities and staff. The average length of the program was 1.42 ± 0.67 days and included 12.16 ± 9.03 individuals.

The lifestyle intervention consisted of the following components.

#### Lecture

A team of physicians, public nurses, fitness trainers, and registered dietitians provided lectures to the participants on health information related to specialized fields (Supplementary Tables 1–3). Public nurses also gave lectures to the participants with regard to understanding the results of their medical checkups, an outline of diabetes including diabetic complications, target to treat, and treatment for diabetes (diet therapy, exercise therapy, and medication). Participants with a BMI > 25 were provided with methods for weight loss. Conversely, participants with a BMI < 25 were advised to maintain a normal BMI.

#### Nutritional intervention

A nutritional intervention based on Dietary Guidelines for the Japanese^22^ and the Japanese Food Guide^23^ was performed by registered dietitians. Registered dietitians gave lectures about a balanced diet, adequate calorie intake, and a low glycemic index diet. They also provided the participants with a BMI > 25 with methods for weight loss, such as by cutting down calorie intake, especially snacks. Participants with a BMI < 25 were advised to maintain an adequate calorie intake and refrain from consuming excessive carbohydrates. The intervention included not only counseling but also practical methods, such as intake of buffet-style meals made from local ingredients. The participants were advised on how to choose a well-balanced meal by comparing it with the meals they actually selected. In some cases, chefs provided lectures on how to cook healthy meals that contain local seasonal ingredients.

#### Exercise

Before the program, fitness trainers evaluated the participants’ physical activity levels using pedometers. They also gave lectures about adequate exercise intensity, methods for walking safely, and self-training at home. A combination of aerobic exercise and muscle resistance training based on the Exercise and Physical Activity Guide for Health Promotion^24^ and Japanese official physical activity guidelines for health promotion (active guide)^25^ was performed. The program included not only counseling but also familiar exercise programs, such as hiking and walking in tourist resorts. The amounts of physical activity and energy expenditure during leisure activities were measured using an activity meter or the Borg scale^26^. The blood glucose levels were measured using a glucometer before and after exercise to help participants realize the effectiveness of exercise.

#### Follow-up support

The participants were followed up by telephone, email, or one-to-one meetings for an average of six times (3–20 times). This was done to motivate the participants to focus on their goals and solve their difficulties in achieving their goals.

### Outcome measures

Based on the national standard of the SHC, body composition parameters included weight (kg), BMI, WC (cm), and SBP and DBP (mmHg), and metabolic parameters included triglycerides (TG; mg/dl), high-density lipoprotein cholesterol (HDL-C; mg/dl), low-density lipoprotein cholesterol (LDL-C; mg/dl), FPG (mg/dl), HbA1c (%), aspartate aminotransferase (AST; U/l), alanine aminotransferase (ALT; U/l), and gamma-glutamyl transferase (ɤ-GTP; U/l). All parameters were measured before and after the program (in 2014 and 2016). WC was measured at the umbilical level. Moreover, lifestyle questionnaires were distributed according to the national standard of SHCs (Supplementary Table 4)^27^. Data collection in both 2014 and 2016 included these questionnaires.

### Sample size

Our pilot study for SHG demonstrated that there was a decrease of 3.14 ± 10.69 in FPG from baseline to 1 year in the intervention group and 1.01 ± 16.28 in the control group^21^. Using Hedge’s *g*, the effect size estimate was calculated as 0.16^28^. The sample size was calculated as 624 participants per group (1248 in total), with a power of 80% and a significance level of 5% (two-tailed). A low dropout rate was estimated as the effectiveness of the program was assessed through mandatory medical checkups.

### Statistical analysis

The missing values for health checkup data and questionnaire responses were complemented by multiple imputation methods using the SPSS missing values option.

In an attempt to decrease the bias between both groups, inverse probability weighting of a propensity score was performed. The propensity score using binary logistic regression analysis was estimated based on sex, age, baseline examination data, and recruitment consortiums (c Statistic 0.85). Laboratory data changes from 2014 to 2016 were analyzed through paired *t*-tests. The differences in the changes between the groups with generalized estimating equations were also analyzed. As for the lifestyle questionnaires, the percentages of people who had a healthy lifestyle and were graded at the action or maintenance phase were compared between 2014 and 2016 using the McNemar test. Moreover, the percentages of people who improved their lifestyle or progressed across the stages of the trans-theoretical model^29^ were also compared between the groups using generalized estimating equations. The average score was substituted for the percentage of people who improved their lifestyle, such that individuals with an improved lifestyle were coded as 1. All statistical analyses were conducted with a significance level of 5% (two-tailed).

## Results

**Fig. 2:**
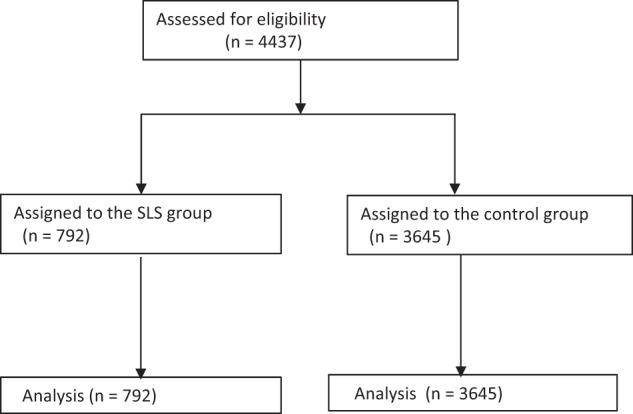
Flow diagram of recruitment, assignment, and 2-year follow-up.

The participants of the program (the SLS group) included 792 individuals, whereas the control group consisted of 3645 (Fig. 2).

Tables 1 and 2 present the baseline values for the SLS group and control group with complementing missing data. The percentages of men, current habitual smoking, physical activity, late dinner, daily drinking, and action phase or maintenance phase were lower in the SLS group, although the percentage of regular exercise was significantly higher compared with that in the control group. As for the medical data, BW, SBP, DBP, FPG, and ɤ-GTP were lower in the SLS group, although age, WC, and LDL-C were significantly higher compared with those in the control group. These significant differences disappeared after adjusting for confounding factors via the inverse probability weighting of a propensity score, except for sex, BW, and current habitual smoking.

**Table 1:** Baseline and 2-year follow-up data related to lifestyle in the SLS and control groups.

Questionnaire	SLS (*n* = 792)		*P*-value	Control (*n* = 3645)		*P*-value
	Baseline	2-year		Baseline	2-year	
Smoking						
Current habitual smoking						
No	664/792	686/792	<0.001***	2478/3645	2572/3645	<0.001***
	83.8%	86.6%		68.0%	70.6%	
Exercise						
Regular exercise						
Yes	230/792	331/792	<0.001***	860/3645	918/3645	0.022*
	29.0%	41.8%		23.6%	25.2%	
Physical activity						
Yes	287/792	352/792	<0.001***	1870/3645	1927/3645	0.037*
	36.2%	44.4%		51.3%	52.9%	
Diet						
Late dinner						
No	525/792	560/792	0.005**	1695/3645	2128/3645	<0.001***
	66.3%	70.7%		46.5%	58.4%	
Snacks						
No	637/792	676/792	<0.001**	2881/3645	2922/3645	0.090
	80.4%	85.4%		79.0%	80.2%	
Skipping breakfast						
No	683/792	658/792	0.008**	3202/3645	3105/3645	<0.001***
	86.2%	83.1%		87.8%	85.2%	
Alcohol						
Daily drinking						
No	627/792	593/792	<0.001***	2307/3645	2268/3645	0.047*
	79.2%	74.9%		63.3%	62.2%	
Trans-theoretical model						
Action or maintenance phase						
Yes	274/792	424/792	<0.001***	1426/3645	1380/3645	0.108
	34.6%	53.5%		39.1%	37.9%	

Questionnaire:

(1) Are you currently a habitual smoker? (“A current habitual smoker” is defined as a person who has smoked a total of 100 cigarettes or more, or has a history of smoking for more than 6 months, and has been smoking for the past one month).

(2) Have you performed exercise with slight sweating for 30 min or more, at least twice a week, for more than 1 year?

(3) Do you walk or engage in some physical exercise equivalent to walking, for 1 h or more a day?

(4) Do you eat dinner within 2 h before sleep at least three times a week?

(5) Do you eat any snacks after dinner (a bed time snack, other than three regular meals) three times or more a week?

(6) Do you miss breakfast three times or more a week?

(7) Do you drink alcoholic beverages every day?

(8) Have you already attempted to improve your lifestyle for <6 months (action phase) or more than six months (maintenance phase) based on the trans-theoretical model?

*SLS* Smart Life Stay program group.

*P-value < 0.05, **P-value < 0.01, ***P-value < 0.001 (baseline vs. 2 years).

**Table 2:** Baseline and 2-year follow-up medical data in the SLS and the control groups.

Continuous variables	SLS (*n* = 792)		*P*-value	Control (*n* = 3645)		*P*-value
	Baseline	2-year		Baseline	2-year	
Sex (male/female)	559/233			3335/310		
Age	53.87 ± 0.37			51.60 ± 0.13		
BW (kg)	71.37 ± 0.51	69.62 ± 0.50	<0.001***	73.02 ± 0.21	73.07 ± 0.21	0.487
BMI (kg/m^2^)	25.83 ± 0.14	25.23 ± 0.15	<0.001***	25.61 ± 0.06	25.66 ± 0.06	0.039*
WC (cm)	89.48 ± 0.35	88.03 ± 0.36	<0.001***	88.67 ± 0.15	89.05 ± 0.16	<0.001***
SBP (mmHg)	126.22 ± 0.55	126.28 ± 0.62	0.922	127.82 ± 0.22	128.52 ± 0.27	0.009**
DBP (mmHg)	78.71 ± 0.41	78.17 ± 0.46	0.152	80.20 ± 0.16	80.70 ± 0.17	0.003**
TG (mg/dl)	143.05 ± 3.40	129.39 ± 3.79	<0.001***	145.72 ± 1.77	145.47 ± 2.08	0.9
HDL-C (mg/dl)	57.25 ± 0.55	59.02 ± 0.56	<0.001***	56.94 ± 0.25	57.93 ± 0.26	<0.001***
LDL-C (mg/dl)	131.64 ± 1.16	126.10 ± 1.16	<0.001***	127.06 ± 0.51	125.51 ± 0.59	0.004**
FPG (mg/dl)	107.46 ± 1.26	107.72 ± 1.03	0.843	111.86 ± 0.45	115.23 ± 0.48	<0.001***
HbA1c (%)	6.09 ± 0.03	6.05 ± 0.03	0.112	6.09 ± 0.01	6.21 ± 0.01	<0.001***
AST (IU/l)	25.77 ± 0.42	24.61 ± 0.53	0.029*	25.42 ± 0.21	25.57 ± 0.31	0.607
ALT (IU/l)	31.89 ± 0.83	27.57 ± 0.79	<0.001***	31.99 ± 0.39	31.20 ± 0.43	0.052
γGTP (IU/l)	48.15 ± 1.62	43.32 ± 1.68	<0.001***	54.09 ± 0.89	54.48 ± 1.05	0.623

Values are mean (SE).

*SLS* Smart Life Stay program group.

*P-value < 0.05, **P-value < 0.01, ***P-value < 0.001 (baseline vs. 2 year).

At the 2-year follow-up, the SLS group had significant improvements on the lifestyle questionnaire items, such as current habitual smoking, regular exercise, physical activity, late dinner, snacks, and movement through stages of the trans-theoretical model. Beneficial changes were also observed in the control group, although the percentage of snacks and trans-theoretical model was unchanged (Table 1). In the comparison between the groups, the percentage of people who had improved their lifestyle habits, such as regular exercise and stage of trans-theoretical model, was significantly higher in the SLS group at the 2-year follow-up (Table 3).

**Table 3:** Change in lifestyle data from baseline to 2-year follow-up in the SLS and control groups.

Questionnaire	Percentage of people who improved lifestyle		*P*-value
	SLS (*n* = 792)	Control (*n* = 3645)	
	(%)	(%)	
Smoking			
Current habitual smoking			
No	7.96 ± 2.73	3.92 ± 0.35	0.139
Exercise			
Regular exercise			
Yes	18.85 ± 3.01	9.46 ± 0.83	0.001**
Physical activity			
Yes	15.10 ± 2.54	11.29 ± 0.73	0.135
Diet			
Late dinner			
No	14.44 ± 2.65	11.53 ± 0.77	0.267
Snacks			
No	11.27 ± 2.29	9.07 ± 0.69	0.33
Skipping breakfast			
No	3.85 ± 1.73	3.52 ± 0.56	0.836
Alcohol			
Daily drinking			
No	5.10 ± 1.80	4.49 ± 0.44	0.726
Trans-theoretical model			
Action or maintenance phase			
Yes	22.91 ± 3.10	11.39 ± 0.84	<0.001***

Values are mean (SE).

Questionnaire:

(1) Are you currently a habitual smoker? (“A current habitual smoker” is defined as a person who has smoked a total of 100 cigarettes or more, or has a history of smoking for more than 6 months, and has been smoking for the past one month).

(2) Have you performed exercise with slight sweating for 30 min or more, at least twice a week, for more than 1 year?

(3) Do you walk or engage in some physical exercise equivalent to walking, for 1 h or more a day?

(4) Do you eat dinner within 2 h before sleep at least three times a week?

(5) Do you eat any snacks after dinner (a bed time snack, other than three regular meals) three times or more a week?

(6) Do you miss breakfast three times or more a week?

(7) Do you drink alcoholic beverages every day?

(8) Have you already attempted to improve your lifestyle for <6 months (action phase) or more than six months (maintenance phase) based on the trans-theoretical model?

*SLS* Smart Life Stay program group.

*P-value < 0.05, **P-value < 0.01, ***P-value < 0.001 (SLS vs. control).

As for the medical data in the SLS group, BW, BMI, WC, TG, LDL-C, AST, ALT, and ɤ-GTP decreased significantly by 1.75 kg, 0.60 kg/m^2^, 1.45 cm, 13.66 mg/dl, 5.54 mg/dl, 1.16 IU/l, 4.32 IU/l, and 4.83 IU/l, respectively. HDL-C increased significantly by 1.770 mg/dl. In the control group, WC, SBP, DBP, FPG, and HbA1c increased significantly by 0.38 cm, 0.70 mmHg, 0.50 mmHg, 3.37 mg/dl, and 0.12%, respectively, although HDL-C and LDL-C improved (Table 2). In the comparison between the groups, the changes in BW, BMI, WC, TG, HDL-C, FPG, HbA1c, AST, ALT, and ɤ-GTP were significantly better in the SLS group at the 2-year follow-up (Table 4).

**Table 4:** Change in medical data from baseline to 2-year follow-up in the SLS and the control groups.

Continuous variables	Changes		*P-*value
	SLS (*n* = 792)	Control (*n* = 3645)	
	Mean ± SE	Mean ± SE	
BW (kg)	−1.52 ± 0.23	−0.01 ± 0.07	<0.001***
BMI (kg/m^2^)	−0.52 ± 0.08	0.03 ± 0.03	<0.001***
WC (cm)	−1.04 ± 0.25	0.30 ± 0.08	<0.001***
SBP (mmHg)	1.08 ± 0.93	0.64 ± 0.28	0.643
DBP (mmHg)	0.11 ± 0.58	0.38 ± 0.18	0.658
TG (mg/dl)	−16.73 ± 5.95	−2.51 ± 2.04	0.026*
HDL-C (mg/dl)	3.38 ± 0.57	0.90 ± 0.18	<0.001***
LDL-C (mg/dl)	−4.90 ± 1.61	−1.99 ± 0.57	0.084
FPG (mg/dl)	−0.08 ± 1.57	3.42 ± 0.51	0.026*
HbA1c (%)	−0.01 ± 0.03	0.11 ± 0.01	0.000**
AST (IU/l)	−1.53 ± 0.61	−0.02 ± 0.33	0.025*
ALT (IU/l)	−4.34 ± 1.01	−1.20 ± 0.48	0.004*
γGTP (IU/l)	−6.14 ± 1.97	0.02 ± 0.81	0.002*

Values are mean (SE).

*SLS* Smart Life Stay program group.

*P-values < 0.05, **P-values < 0.01, ***P-values < 0.001.

## Discussion

The SLS program is an experience-oriented stayover program for people with diabetes or at a high risk of diabetes. This program was conducted using existing multicenters, such as resort hotels, community centers, and spas. It was also supported by health-care professionals, other than medical institutions. A wide variety of programs were developed throughout the nation, and the programs were combined with various practical programs supplied from local resources based on SHG.

The SLS program improved the lifestyle of the participants; consequently, BMI, WC, FPG, and HbA1c were significantly reduced compared with the control group. This program also significantly improved other metabolic parameters compared with the control group, such as TG, HDL-C, AST, ALT, and ɤ-GTP.

The effect of this program on weight reduction and glucose metabolism was nearly equal to that of other lifestyle interventions in the real-world setting^30–32^. The duration and frequencies of this intervention program were comparatively shorter and less than that of other intervention programs. In addition, the follow-up period of this program (i.e., 2 years), was longer than that in other studies, thus indicating the importance of “experience-oriented” intervention programs.

The most unique characteristic of this program was the use of the existing database. In previous intervention trials, setting the control group was difficult. However, it was comparatively easy to set the control group by using an existing database of nationwide uniform medical checkups in Japan. Currently, lifestyle intervention programs must be performed using existing facilities and databases because of their feasibility. Thus, the success of the study is significant.

This study had some limitations, including the selection bias that might have resulted from the lack of randomization. Controls were set using the inverse probability weighting for the purpose of adjusting confounding factors to maintain selection bias at a minimum. Another limitation was the possibility that the participants in both the SLS group and control group received additional lifestyle interventions as people are comparatively easy to access health information and health guidance in Japan. Third, the contents of the program lacked unity and were determined individually by each recruitment medical insurer, except for the core program. Conversely, each program might have had regional characteristics, because program flexibility was permitted.

## Conclusions

This study was conducted to investigate the effectiveness of a health tourism program for individuals with diabetes and those at a high risk of diabetes. Currently, to perform lifestyle interventions in a real-world setting, an effective use of existing facilities and databases is required. This program might be a feasible option for a lifestyle intervention program for patients with diabetes and those at a high risk of diabetes. In the future, this program has the potential to improve the health of participants and to provide economic benefits to local communities.

## Supplementary information

Supplemental Table 1

Supplemental Table 2

Supplemental Table 3

Supplemental Table 4

## References

[1] International Diabetes Federation. *IDF Diabetes Atlas 2019*. https://www.diabetesatlas.org/en/ (2019).

[2] World Health Organization. *Global Report on Diabetes 2016*. www.who.int/diabetes/global-report. Accessed 16 Aug 2018 (2016).

[3] Pan XR (1997). Effects of diet and exercise in preventing NIDDM in people with impaired glucose tolerance. The Da Qing IGT and Diabetes Study. Diabetes Care..

[4] Knowler WC (2002). Reduction in the incidence of type 2 diabetes with lifestyle intervention or metformin. N. Engl. J. Med..

[5] Lindstrom J (2006). Sustained reduction in the incidence of type 2 diabetes by lifestyle intervention: follow-up of the Finnish Diabetes Prevention Study. Lancet.

[6] Muramoto A (2014). Three percent weight reduction is the minimum requirement to improve health hazards in obese and overweight people in Japan. Obes. Res Clin. Pract..

[7] Muramoto A (2010). Effect of intensive lifestyle intervention programs on metabolic syndrome and obesity: how much weight reduction is needed to improve metabolic comorbidities?. J. Jpn. Soc. Study Obes..

[8] Nakamura T (2013). The relationship between change of the amount of exercise or energy intake and change of medical data. Tokai J. Public Health.

[9] Moriguchi J (2011). Effectiveness of specific health guidance against metabolic syndrome. Off. J. Jpn. Soc. Ningen Dock..

[10] Haruyama Y, Muto T, Nakate M, Yamasaki A, Tarumi F (2012). Changes in measurements related to metabolic syndrome among individuals with national health insurance after specific health guidance. Jpn. J. Public Health.

[11] Ishikawa Y, Imai H, Nakao H, Saito K, Fukuda Y (2013). Research about the effectiveness of specific health guidance: casual relation analysis of large scale data using propensity score. J. Health Welf. Stat..

[12] Ministry of Health, Labor, and Welfare in Japan. *The Final Reports of the 14th Working Group for Evaluating the Effectiveness for Setting Reasonable Medical Charges of Specific Health Check-ups and Specific Health Guidance*. http://www.mhlw.go.jp/file/05-Shingikai-12401000-Hokenkyoku-Soumuka/0000090330.pdf. Accessed 16 Aug 2018.

[13] Chan JC (2009). Diabetes in Asia: epidemiology, risk factors, and pathophysiology. JAMA.

[14] Ray P. H., Anderson S. R. *The Cultural Creatives: How 50 Million People are Changing the World* (Broadway Books, 2001).

[15] Des Jarlais DC, Lyles C, Crepaz N, Group T (2004). Improving the reporting quality of nonrandomized evaluations of behavioral and public health interventions: the TREND statement. Am. J. Public Health.

[16] Seino Y, Committee of the Japan Diabetes Society on the Diagnostic Criteria of Diabetes M (2010). Report of the committee on the classification and diagnostic criteria of diabetes mellitus. J. Diabetes Investig..

[17] Inzucchi SE (2012). Management of hyperglycemia in type 2 diabetes: a patient-centered approach: position statement of the American Diabetes Association (ADA) and the European Association for the Study of Diabetes (EASD). Diabetes Care..

[18] Simons FE (2015). 2015 update of the evidence base: World Allergy Organization anaphylaxis guidelines. World Allergy Organ J..

[19] APA. *Diagnostic and Statistical Manual of Mental Disorders (DSM-5)* 490–503 (American Psychiatric Association, 2013).

[20] APA. *Diagnostic and Statistical Manual of Mental Disorders (DSM-5)* 591–643 (American Psychiatric Association, 2013).

[21] Tsushita K (2018). Rationale and descriptive analysis of specific health guidance: the nationwide lifestyle intervention program targeting metabolic syndrome in Japan. J. Atheroscler. Thromb..

[22] Ministry of Agriculture, Forestry and Fisheries in Japan. *Dietary Guidelines for Japanese 2016*. http://www.maff.go.jp/j/syokuiku/attach/pdf/shishinn-3.pdf. http://www.maff.go.jp/j/syokuiku/attach/pdf/shishinn-5.pdf. Accessed 4 Oct 2018.

[23] Ministry of Health, Labor, and Welfare and Ministry of Agriculture, Forestry and Fisheries in Japan. *Japanese Food Guide Spinning Top*. http://www.maff.go.jp/j/balance_guide/b_use/pdf/eng_reiari.pdf. Accessed 4 Oct 2018.

[24] Ministry of Health, Labor, and Welfare in Japan. *Exercise and Physical Activity Guide for Health Promotion*. http://www.nibiohn.go.jp/eiken/programs/pdf/exercise_guide.pdf. Accessed 4 Oct 2018.

[25] Ministry of Health, Labor, and Welfare in Japan. *Japanese Official Physical Activity for Health Promotion*. http://www.nibiohn.go.jp/eiken/info/pdf/active2013-e.pdf. Accessed 4 Oct 2018.

[26] Borg GA (1982). Psychophysical bases of perceived exertion. Med Sci. Sports Exerc..

[27] Ministry of Health, Labor, and Welfare in Japan. *Standard Program for the Specific Health Check-ups*. http://tokutei-kensyu.tsushitahan.jp/manage/wp-content/uploads/2014/05/36ec0bcdf91b61a94a1223627abffe8d.pdf. Accessed 4 Oct 2018.

[28] Lakens D (2013). Calculating and reporting effect sizes to facilitate cumulative science: a practical primer for t-tests and ANOVAs. Front. Psychol..

[29] Prochaska JO, Velicer WF (1997). The transtheoretical model of health behavior change. Am. J. Health Promot..

[30] Vuksan V (2017). Salba-chia (*Salvia hispanica* L.) in the treatment of overweight and obese patients with type 2 diabetes: a double-blind randomized controlled trial. Nutr. Metab. Cardiovasc. Dis..

[31] Kulzer B, Hermanns N, Gorges D, Schwarz P, Haak T (2009). Prevention of diabetes self-management program (PREDIAS): effects on weight, metabolic risk factors, and behavioral outcomes. Diabetes Care..

[32] Absetz P (2009). Type 2 diabetes prevention in the real world: three-year results of the GOAL lifestyle implementation trial. Diabetes Care..

