# Correction: The Smart Life Stay (SLS) program: effects of a lifestyle intervention program in combination with health tourism and health guidance for type 2 diabetes

**DOI:** 10.1038/s41387-020-00137-w

**Published:** 2020-09-23

**Authors:** Madoka Matsushita, Akiko Muramoto, Eri Nomura, Yukari Eguchi, Ayako Kato, Yoshiko Sano, Mai Kabayama, Masashi Arakawa, Yuko Oguma, Daisuke Yabe, Masaaki Matsunaga, Hiroshi Yatsuya, Hiroshi Arima, Kazuyo Tsushita

**Affiliations:** 1 Comprehensive Health Science Center, Aichi Health Promotion Public Interest Foundation, 1-1, Gengoyama, Morioka, Higashiura-cho, Chita-gun, Aichi-ken 470-2101 Japan; 2 Department of Endocrinology and Diabetes, Nagoya University Graduate School of Medicine, 65 Tsurumai-cho, Shouwa-ku, Ngoya-shi, Aichiken 466-8550 Japan; 3 Kanagawa University of Human Services, Faculty of Health & Social Services, School of Nutrition & Dietetics, 1-10-1 Heisei-cho, Yokosuka-shi, Kanagawa-ken 238-8522 Japan; 4 Division of Health Sciences, Osaka University Graduate School of Medicine, 1-7 Yamadaoka, Suita-shi, Osaka-fu 565-0871 Japan; 5 Health Tourism Research Fields, Graduate School of Tourism Sciences University of the Ryukusu, 1 Senbaru, Nishihara-cho, Nakagami-gun, Okinawaken 903-0129 Japan; 6 Sports Medicine Research Center & Graduate School of Health Management, Keio University, 4-1-1 Hiyoshi, Kohoku-ku, Yokohama, Kanagawa-ken 223-8521 Japan; 7 Department of Diabetes and Endocrinology, Gifu University Graduate School of Medicine, 1-1 Yanagido, Gifu-shi, Gifu-ken 501-1194 Japan; 8 Department of Public Health, Fujita Health University School of Medicine, 1-98 Dengakugakubo, Kutsukake-cho, Toyoake-shi, Aichi-ken 470-1192 Japan

**Keywords:** Preventive medicine, Clinical trials

Correction to: *Nutrition & Diabetes*

10.1038/s41387-020-00136-x published online 29 August 2020

Following publication, the authors asked to add the following institution to the affiliations of author Madoka Matsushita:

Department of Endocrinology and Diabetes, Nagoya University Graduate School of Medicine

Both the PDF and HTML versions of the Article have been updated accordingly.

